# Patterns and Determinants of Family Support among Pregnant Women in Ile-Ife, Nigeria: A Quantitative Cross-Sectional Study

**DOI:** 10.4314/ejhs.v34i3.9

**Published:** 2024-05

**Authors:** Love Bukola Ayamolowo, Esther Adebola Adekunle, Sunday Joseph Ayamolowo, Bukola Abimbola Adesoji, Phebian Funmilayo Adekunle

**Affiliations:** 1 Department of Nursing Science, Obafemi Awolowo University, Nigeria; 2 Department of Nursing Services, Obafemi Awolowo University Teaching Hospital Complex, Nigeria; 3 Department of Physical and Health Education, Obafemi Awolowo University, Nigeria

**Keywords:** Family, support, pregnant mothers, antenatal

## Abstract

**Background:**

Effective family support is essential for promoting the well-being of pregnant women and reducing the risk of adverse pregnancy outcomes. This study examined family support patterns and influencing factors among pregnant women attending antenatal clinics in Ile-Ife, Nigeria.

**Methods:**

This descriptive cross-sectional study involved 384 pregnant women in a Local Government Area in southwestern Nigeria. Three healthcare facilities with the highest antenatal attendance were selected through purposive sampling. Data were collected using a tested and structured questionnaire, which was developed following a review of similar studies. The results were analyzed using SPSS version 20.0, employing Pearson Chi-square tests with a significance level set at p < 0.05.

**Results:**

More than half of the respondents reported inadequate family support in various aspects including tangible/instrumental support (52.9%), emotional support (51.4%), and financial support (54.4%). However, the majority reported significant financial supports from their spouses (60.7%). Religion, educational qualification, and partner's occupation were found to significantly influence the likelihood of women receiving higher levels of social support

**Conclusion:**

Many respondents lacked adequate support from spouses and families. Encouraging family involvement in antenatal care can improve understanding and support, benefiting maternal and child health. Hence, healthcare professionals and policymakers should consider the factors influencing family support options when designing focused interventions to strengthen maternal support systems and address the varied needs of pregnant women.

## Introduction

Pregnancy is a transformative period characterized by a multitude of physical and psychological changes (1). Expectant mothers often find themselves navigating a range of new circumstances and emotions, requiring attention and support during this critical period (2). The adjustments and challenges during pregnancy can significantly influence a pregnant woman's attitudes, decision-making, and overall behaviour as she assumes the responsibilities of impending motherhood (3). Notably, social support plays a pivotal role in alleviating the stress associated with pregnancy and promoting positive maternal and newborn health outcomes (4).

Social support encompasses the provision of emotional, informational, instrumental, tangible, and psychological support by the social network of family members, friends, and the community (5,6). It serves as a vital foundation upon which pregnant women can lean, helping them navigate the intricacies of pregnancy with greater ease. Pregnant women who have been well-buttressed by their family and social network tend to be less frequently affected by psychological problems, such as distress, anxiety disorders, and depression (6). Conversely, inadequate social support during pregnancy has been associated with mental health challenges and negative birth outcomes for expectant mothers (7,8). Inadequate social support poses a notable risk for depression, with maternal mental health being significantly impacted by stress and the quality of relationships (9,10). Additionally, factors such as domestic violence, poverty, and inadequate social support have been associated with a higher prevalence of maternal depression (11).

Promoting social support systems for pregnant women is vital for their emotional, physical, and overall well-being, ultimately leading to healthier pregnancies and positive outcomes for both mothers and children (12). This study looked into the intricate dynamics of social support during pregnancy and its potential significance in providing valuable insights to healthcare professionals, particularly midwives and policymakers, regarding the multifaceted nature of social support during pregnancy. Armed with a deeper understanding of the determinants identified in this study, midwives can develop strategies to enhance maternal well-being and improve maternal and child health outcomes. Furthermore, this research aligns with the broader goal of advancing maternal and child health in Nigeria, a nation still grappling with unacceptably high maternal mortality rates (13).

With family support being a cornerstone of maternal and child health, this study's primary aim was to assess the key determinants of family support for pregnant women attending antenatal clinics in Ile-Ife, Nigeria. Identifying these influential factors allows for targeted interventions and policies aimed at strengthening the support systems available to expectant mothers. These interventions have the potential to alleviate the burden of maternal mental health issues, reduce adverse birth outcomes, and contribute to overall improvements in maternal and child health.

## Materials and Methods

**Study area, design and population**: This is a cross-sectional study conducted from January to March, 2023, among pregnant women attending antenatal clinics in Ile-Ife, Nigeria. Ile-Ife has a smaller population compared to major urban centres like Lagos, Kano, and Ibadan in Nigeria. The city combines both rural and urban characteristics, with an urban city centre and surrounding rural areas characterized by traditional lifestyles, agriculture, smaller settlements, and strong adherence to local customs. The gender distribution in the city is roughly balanced between males and females, with a slightly higher proportion of females due to cultural and demographic factors. It is made up of two Local Government Areas (LGAs): Ife Central and Ife East, each equipped with public healthcare facilities.

In these healthcare facilities, doctors, nurses and midwives actively deliver quality antenatal care to pregnant mothers. The provision of quality care is further supported by community healthcare workers, especially in the rural areas.

**Target population and sampling**: This study was targeted at pregnant women within the ages of 15-49 years attending antenatal clinics in the selected LGA (Ife Central Local Government). One hospital with the highest antenatal attendance was purposefully chosen from each level of the healthcare delivery system to ensure an adequate number of respondents for the study. The sample size for this study was calculated using Cochran's formula: N (minimum sample size) = Z^2 * (P*Q)/D^2. A prevalence of family support option (50%) was used. With the P-value set at 5% confidence interval, a minimum sample size of 384 was estimated; however, a non-response rate of 10% was added. Hence, the total sample size was 422. The total sample size was divided by the average number of pregnant women attending the clinic weekly to estimate the sampling interval. Participants were selected at random every week until the desired sample size was achieved. Pregnant women that were not physically present and women with pregnancy complications were excluded from the study. Women eligible for the study were recruited after obtaining their consent.

**Data collection instrument**: Data were collected using a semi-structured questionnaire adapted from literatures (14,15) that were reviewed, and it was subsequently modified to align with the specific objectives of the research. The items of the questionnaire was used to elicit information about socio-demographic characteristics, obstetric characteristics, and pattern of family support options among respondents. The instrument was reviewed by experts in public health and maternal and child health. The items were ascertained to be relevant to the scope and objectives of the study.

Data were collected using a random sampling technique. Every n^th^ woman on the clinic register was chosen based on the average clinic attendance. The principal investigator and two trained research assistants administered the questionnaire to the pregnant mothers after gaining their consents and providing clarifications. The questionnaire was translated into the commonly understood language for broader accessibility for the women that cannot read the English version of the questionnaire.

**Data analysis**: The responses were coded and analyzed using IBM Statistical Product of Service Solution (SPSS) version 20.0. Descriptive statistical methods such as frequency, tables and percentages were used to analyze univariate statistics, and p-value, odd ratio and confidence interval were used to assess the relationship between the variables The normality of data distribution was determined. The median score (55) derived from result analysis was used to categorize respondents. Those with a median score of 55 and above were classified as having adequate family support, while those below the median score were considered to have inadequate family support.

**Ethical considerations**: The study was reviewed and approved by the Health Research Ethical Committee of the Institute of Public Health, Obafemi Awolowo University, Ile-Ife, Nigeria (IPH/OAU/12/2175). Signed informed consent was obtained from eligible women who agreed to be enrolled in the study.

## Results

A total of 422 copies of the questionnaires were administered, but 384 were completely filled and found suitable for analysis, yielding a response rate of 90.9%. The average age of the participants was 28.8 ± 4.37 years, with more than half (57.5%) falling within the age range of 25-30 years (Table 1). The majority (90.4%) of the respondents were married in a monogamous family setting (63.5%) and identified as Christians (69.3%) and Yoruba tribe (74.2%). More than half (57.3%) of the respondents had attained secondary school education as their highest qualification. Additionally, most of their partners had completed tertiary education (66.7%).

**Table 1:** Socio-demographic characteristics of respondents (N=384)

Variable	Frequency (%)
**Age (Mean = 28.8** ± 4.37)	
Less than 25 years	51(13.3)
25 – 20 years	221(57.5)
30 – 35 years	91(23.7)
More than 35 years	21(5.5)
**Marital Status**	
Single	28(7.3)
Married	117(30.5)
Divorced	9(2.3)
**Religion**	
Christianity	266(69.3)
Islamic	117(30.5)
Traditional	1(0.2)
**Ethnicity**	
Yoruba	285(74.2)
Hausa	63(16.4)
Igbo	33(8.6)
Others	3(0.8)
**Family Setting**	
Polygamous	140(36.5)
Monogamous	284(63.5)
**Educational Qualification**	
None	26(6.8)
Primary	48(12.5)
Secondary	90(23.4)
Tertiary	220(57.3)
**Partner's Educational Qualification**	
None	12 (3.1)
Primary	24 (6.3)
Secondary	75 (19.5)
Tertiary	256 (66.7)
No Response	17 (4.4)
**Occupation**	
Employed	142 (36.9)
Unemployed	101 (26.3)
Self-employed	141 (36.7)
**Partner's Occupation**	
Employed	162 (42.2)
Unemployed	49 (12.8)
Self-employed	173 (45.0)
**Residential Address**	
Rural	153 (39.8)
Urban	231 (60.2)

As shown in Table 2, the majority (80.7%) had experienced between 1 to 3 pregnancies, deliveries (69.3%), and had 1 to 3 children (60.9%). An overwhelming majority (88.5%) of the respondents had never undergone abortion. More than half (58.1%) had 1 to 3 antenatal care visits; the majority had hospital delivery (79.8%) with only 26.7% of them delivering via a caesarean section.

**Table 2:** Obstetrics characteristics of respondents (N=384)

Variables	Frequency (%)
**Number of Pregnancies**	
1 – 3	310(80.7)
4 – 6	65(16.9)
>6	9(2.3)
**Number of Deliveries**	
None	107(27.1)
1 – 3	265(69.3)
4 – 6	12(3.6)
**Number of Children**	
None	128(33.3)
1 – 3	234(60.9)
4 – 6	22(5.7)
**Number of Abortions**	
None	340(88.5)
1 – 3	43(11.5)
4 – 6	1(0.3)
**Mode of Delivery of Last Baby (n=277)**	
Vaginal Delivery	203(73.3)
Caesarean Section	74(26.7)
**Place of Delivery of Last Baby (n-277)**	
Church	12(4.3)
Home	44(15.9)
Hospital	221(79.8)
**Present Gestational Age**	
Less than 20 weeks	113(29.4)
20 – 30 weeks	155(40.4)
More than 30 weeks	116(30.2)
**Antenatal Visits Had**	
1 – 3 visits	223(58.1)
4 – 6 visits	114(29.4)
More than 6 visits	47(12.2)

**Figure 1:**
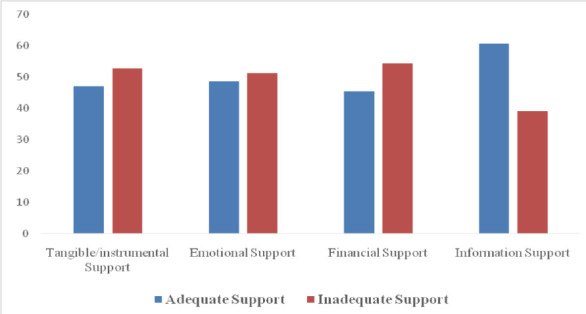
Summary of each level of support options respondents received

Approximately half of the respondents (51.6%) strongly agreed that their partners and other family members help them with household chores. Less than half strongly agreed that they receive support from their partners when confined to bed (46.4%), partner assists in preparing meals when they were unable to do it themselves (43.8%), and a smaller proportion agreed that their partners accompany them during antenatal visits (33.4%). Figure 1 summarizes the various patterns of support available for the pregnant women. A considerable number of respondents reported receiving inadequate levels of support from their spouses in terms of instrumental (52.9%), emotional (51.4%), and informational support (54.4%). However, the majority of the respondents (60.7%) reported receiving a high level of financial support from their spouses (Table 3). The overall level of support in this study shows that more than half of the respondents (50.3%) reported inadequate support options, while 49.7% found their support options adequate.

**Table 3:** Support options of respondents (N=384)

Items	*S/A	A	D	S/D
**Tangible/Instrumental Support**				
My partner and other family members assist me with my home chores	198 (51.6)	134 (34.9)	46 (12)	06 (1.6)
When I am confined in bed, I have the support of my partner	178 (46.5)	156 (40.6)	40 (10.4)	10 (2.6)
My partner accompanies me during antenatal visits	110 (28.6)	118 (30.7)	128 (33.4)	28 (7.3)
My partner assists in preparing meals when I'm unable to do so myself	168 (43.8)	132 (34.4)	78 (20.3)	06 (1.6)
**Emotional Support**				
I have people to count on and listen when I need to talk	228 (59.4)	116 (30.2)	40 (10.4)	-
I can confide in my partner with my problems	210 (54.7)	120 (31.3)	42 (10.9)	12 (3.1)
I can always turn to my partner and other family members for suggestions to deal with a personal problem	184 (48.4)	136 (35.4)	58 (15.1)	04 (1)
I have someone I can share my worries and anxiety with	206 (53.6)	146 (38)	28 (7.3)	04 (1)
My partner makes me feel loved and wanted	206 (53.6)	136 (35.4)	34 (8.9)	8 (2.1)
My partner shows me love and affection	194 (50.5)	148 (38.5)	36 (9.4)	06 (1.6)
**Material/Financial Support**				
I receive financial support from my partner	218 (56.8)	116 (30.2)	36 (9.4)	14(3.6)
I receive financial support from other family members	206 (53.6)	144 (37.5)	20 (5.2)	14(3.6)
I have people that support with the materials needed during pregnancy and childbirth	164 (42.7)	170 (44.3)	36 (9.4)	14(3.6)
**Information Support**				
I have someone that gives me information about my pregnancy	222 (57.8)	116 (30.4)	44 (11.5)	02 (0.5)
Healthcare workers provide me with the necessary information I need	246 (64.1)	124 (32.3)	12 (3.1)	02 (0.5)
My family members give me good advice about my pregnancy care	196 (51)	158 (41.1)	24 (6.3)	06 (1.6)

* S/A= Strongly Agree; A= Agree; D=Disagree, S/D =strongly disagree

Table 4 shows the relationship between sociodemographic characteristics and level of support options among the respondents. Age (χ^2^ = 8.679, OR= 0.189, p= 0.034), marital status (χ^2^ = 10.217, OR= 1.906, p= 0.006), ethnicity (χ^2^ = 30.508, OR= 0.346, p= <0.001), religion (χ^2^= 14.944, OR= 6.717, p= <0.001), family setting (χ^2^ = 5.942, OR= 1.474, p= 0.001), educational qualification (χ^2^= 36.461, OR= 7.778, p= 0.000), occupation of the respondent's (χ^2^ = 23.668, OR= 0.4.219, p= <0.001), occupation of the partner (χ^2^ = 50.199, OR= 2.725, p= <0.001), and place of residence (χ^2^ = 24.714, OR= 0.316, p= <0.001) were significantly associated with support levels received. Obstetric characteristics (Table 5), such as the number of pregnancies (χ^2^= 12.379, OR= 2.073, p = 0.002), number of deliveries (χ^2^= 16.442, OR= 3.621, p= 0.002), number of children (χ^2^= 9.018, OR= 0.890, p= 0.01), and gestational age of respondents (χ^2^= 19.505, OR= 2.546, p= <0.001) were found to be significantly associated with level of support received. The odds ratios indicate significant associations between various factors and the level of support received. Educational qualification emerges as the strongest predictor, with individuals having higher qualifications being nearly eight times more likely to receive increased support. Additionally, religious affiliation, partner's occupation, number of deliveries, and gestational age of respondents all show notable impacts on the level of support received, highlighting the multifaceted nature of social support networks in obstetric care.

**Table 4:** Multivariate analysis of the relationship between sociodemographic characteristics and level of support

Sociodemographic Characteristics	Support Options		χ^2^ (p-value)	OR	95% CI

	Inadequate(n%)	Adequate(n%)			
**Age**					
< 25 years	17 (33.3)	34 (66.7)	8.679	RC	1
25-30 years	122 (55.7)	99 (44.8)	(0.034*)	0.189	0.058 – 0.617
31-35 years	51 (56.0)	40 (44.0)		0.329	0.091 – 1.197
> 35 years	12 (57.1)	9 (42.9)		---	---
**Marital Status**					
Single	16 (57.1)	12 (42.9)	10.217 (0.006*)	RC	1
Married	186 (53.6)	161 (46.4)		1.906	0.234 – 15.557
Divorced	0 (0.0)	9 (100.0)		---	---
**Religion**					
Christianity	157 (59.0)	109 (41.0)	14.944 (<0.001*)	RC	1
Islamic	44 (37.4)	73 (62.4)		6.714	2.852 – 15.819
Traditional	1 (100.0)	0 (0.0)		---	---
**Ethnicity**					
Yoruba	143 (50.2)	142 (49.8)		RC	1
Hausa	26 (41.3)	37 (58.7)	30.508 (<0.001*)	0.036	0.900 – 1.327
Igbo	32 (97.0)	1 (3.0)		0.032	0.200 – 0.518
Others	1 (33.3)	2 (66.7)			
**Family Settings**					
Polygamous	62 (44.3)	78 (55.7)	5.942 (0.015*)	RC	1
Monogamous	140 (57.4)	104 (42.6)		1.474	0.682 – 3.188
**Educational Qualification**					
None	18 (69.2)	8 (30.8)		RC	1
Primary	10 (20.8)	38 (79.2)	36.461 (<0.001*)	7.778	1.340 – 45.144
Secondary	35 (38.2)	55 (61.1)		1.059	0.137 – 8.207
Tertiary	139 (63.2)	81 (36.8)		0.439	0.058 – 3.310
**Partner's Educational Qualification**					
None	4 (33.3)	8 (66.7)		RC	1
Primary	10 (41.7)	14 (58.3)		0.027	0.003 – 0.231
Secondary	38 (50.7)	37 (49.3)	5.128	0.068	0.006 – 0.763
Tertiary	145 (56.7)	111 (43.3)	(0.063)	0.417	0.044 – 4.094
No Response	5 (29.4)	12 (70.6)		0.311	0.021 – 4.542
**Occupation**					
Employed	95(68.5)	44 (31.6)		RC	1
Unemployed	36 (35.6)	65 (64.4)	23.668 (<0.001*)	4.219	1.065 – 16.719
Self-employed	70 (50.0)	70 (50.0)		4.009	1.721 – 9.343
**Partner's Occupation**					
Employed	119 (73.5)	43 (26.5)			1
Unemployed	9 (40.9)	13 (59.1)	50.199 (<.001*)		1.065 – 16.719
Self-employed	69 (39.8)	104 (60.2)			4.027 – 19.671
**Place of Residence**					
Rural	57 (37.3)	96 (62.7)	24.714(<0.001*)	RC	1
Urban	145 (63.6)	83 (36.4)		0.316	0.147 – 0.679

**Table 5:** Multivariate Analysis of the relationship between Obstetric Characteristics and Support level

Sociodemographic Characteristics	Support Options		χ^2^ (p-value)	OR	95% CI

	Inadequate (n%)	Adequate (n%)			
**Number of pregnancies**					
1-3	172 (55.2)	138 (44.5)	12.379	RC	1
4 – 6	30 (46.2)	35 (53.8)	(0.002*)	2.073	0.898 – 4.783
>6	0 (0.0)	9 (100.0)		---	---
**Number of deliveries**					
None	70 (65.4)	36 (34.6)		RC	1
1 – 3	122 (46.0)	143 (54.0)	16.44	2.636	0.968 – 7.177
4 – 6	10 (83.3)	2 (16.7)	90.002*)	3.621	1.042 – 6.261
**Number of Children**					
1 – 3	81 (63.3)	47 (36.7)		RC	1
4 – 6	111 (47.4)	123 (52.6)	9.018	0.890	0.345 – 2.229
> 6	10 (45.5)	12 (54.5)	(0.011*)		---
**Number of abortions**					
None	183 (53.8)	157 (46.2)		RC	
1 – 3	18 (42.9)	24 (57.1)	2.910	0.803	0.362 – 1.777
4 – 6	0 (0.0)	1 (100)	(0.233)		
**Mode of delivery of last baby**					
Vaginal Delivery	98 (48.3)	105 (51.4)	0.118	RC	1
Caesarean Section	34 (45.9)	40 (54.1)	(0.731)	0.857	0.276 – 1.248
**Gestational Age**					
Less than 20 weeks	70 (45.8)	83 (54.2)		RC	1
20 – 30 weeks	57 (61.3)	36 (38.7)	19.505	2.546	1.277 – 1.041
More than 30 weeks	5 (16.1)	26 (83.9)	(>0.001*)	0.849	0.369 – 1.955
**Antenatal visits**					
1 – 3	114 (51.1)	109 (48.9)		RC	1
4 – 6	68 (59.6)	46 (40.4)	4.731	0.537	0.277 – 1.041
>6	20 (57.4)	27 (57.4)	(0.112)	1.709	0.651 – 4.491

* Significant when p-value <0.05, OR=Odds ratio, RC=Reference category, CI=Confidence interval

## Discussion

The study delves into the multifaceted landscape of support dynamics among pregnant women, unveiling critical demographic and reproductive characteristics that shape these dynamics. A considerable segment of respondents falls within the pivotal reproductive age of 25 to 30 years, closely tied to heightened fertility rates in Nigeria (9,10). Marital status and family settings exerted significant influences, with those in monogamous marriages experiencing different support dynamics than their polygamous counterparts, who may grapple with feelings of loneliness and interpersonal insensitivity (11). Interestingly, the study nuances this, acknowledging complexities in support within polyamorous relationships (12). The socio-economic tapestry varies, reflecting diverse educational backgrounds, with many completing secondary education, while partners often hold tertiary qualifications. Employment status introduces an economic layer, where less than half of the respondents are employed, and potentially influencing support dynamics (16). Urban residence, predominant among participants, emphasizes the urban-rural divide in accessing support systems (17). In tandem, reproductive characteristics underscore nuanced realities. Obstetric experiences, such as the number of pregnancies, deliveries, and children as well as antenatal visits, mirror global fertility trends, revealing a comprehensive snapshot of the participants' reproductive history (18,19). This variance necessitates tailored interventions based on individual reproductive profiles, improved antenatal and postnatal care, and targeted support to enhance overall maternal and child well-being.

The intricate dance of social and emotional support further unfolds, revealing a dichotomy where respondents express concerns and stress to partners and family members, finding solace, yet grappling with occasional feelings of overwhelm from excess attention (10). Different dimensions of support, including instrumental, emotional, informational, and financial support, weave a complex tapestry (22). While certain aspects such as aid with household chores and meal preparation garner significant consensus, there is less unanimity regarding accompaniment to antenatal visits. These visits are recognized as vital components of obstetric care, widely acknowledged as the most crucial measure for mitigating maternal mortality rates (23). Emotional support emerges as a robust cornerstone in this context, often complemented by financial assistance (24).

Informational support primarily flows from health workers, family members, and partners, cementing their role as key sources of pregnancy-related information (25,26,27). The study uncovers disparities, indicating significant financial support from spouses but gaps in emotional and informational support. Interventions should address these discrepancies, fortifying that holistic support networks will not only improve maternal well-being but also contribute to healthier outcomes for both mothers and their children.

Marital status emerges as a pivotal determinant, influencing support options, with divorced pregnant women facing diminished likelihood of adequate support compared to single or married counterparts. This indicates the necessity of tailored interventions to provide enhanced support for pregnant women, especially those who are divorced, ensuring they receive the necessary assistance and care during this critical period. Employment status also has a positive influence, correlating with higher support levels, highlighting the intricate interplay of social and economic factors in shaping support networks (28). These findings underscore the need for economically inclusive support programs to ensure equitable access during pregnancy, contributing to enhanced maternal and child health outcomes.

Social support plays a vital role in alleviating stress and enhancing the emotional and physical health of pregnant mothers. Pregnant women lacking sufficient social support are susceptible to substance use, increased vulnerability to mental health issues, and negative birth outcomes (6). Understanding the factors associated with social support is crucial in developing effective interventions to enhance social support systems for pregnant mothers, promoting healthier pregnancies and outcomes for both the mother and the child. Midwives, as primary caregivers, must navigate these nuances, ensuring that adequate support systems are in place for pregnant women. The study, in its richness and depth, opens avenues for targeted interventions, emphasizing the need for healthcare providers and policymakers to craft nuanced support programs that acknowledge and address the diverse determinants shaping the support landscape for pregnant women in Ile-Ife, Nigeria.

In conclusion, the study shows that many pregnant women experienced lack of adequate support from spouses and families. Financial support from spouses or partners was reported as high, while other pattern of support, including instrumental, emotional, and informational support from spouses were noted as relatively low. Marital status, occupation, parity, and partner's occupation were identified as significant factors shaping these disparities in support levels. Hence, healthcare professionals and policymakers should consider these various influences when designing targeted interventions to strengthen maternal support systems and meet the diverse needs of pregnant women.
